# Irregularity in Daily Activities Predicts Depression via Reduced Perceived Control: A Daily Diary Study

**DOI:** 10.1002/jclp.70124

**Published:** 2026-03-02

**Authors:** Jaeyeon Jang, Sunkyung Yoon

**Affiliations:** ^1^ Department of Psychology Sungkyunkwan University Seoul South Korea

**Keywords:** mental health, regular lifestyle, routinization, sense of control, social rhythm

## Abstract

**Objectives:**

Disruptions in social rhythm—referring to irregularities in daily activities such as meals, work, and social interactions—have been associated with increased psychological distress, including depression. While circadian rhythm‐related factors (e.g., sleep quality) have been proposed as key mechanisms underlying this link, emerging evidence points to additional psychological pathways. This study aimed to examine perceived control as a potential psychological mediator between social rhythm irregularity and depression.

**Methods:**

A 14‐day daily diary study was conducted with 124 participants. We investigated the mediating role of perceived control in the relationship between social rhythm irregularity and depression at both the between‐person and within‐person levels.

**Results:**

At the between‐person level, lower perceived control significantly mediated the association between greater social rhythm irregularity and higher depressive symptoms, even after controlling for sleep quality as a parallel mediator. At the within‐person level, results from a 1‐1‐1 multilevel mediation model showed that daily perceived control fully mediated the link between daily social rhythm irregularity and end‐of‐day depressive affect.

**Conclusion:**

These findings underscore the importance of considering psychological mechanisms, such as perceived control, alongside circadian rhythm‐related factors when examining the mental health consequences of irregular daily routines.

Daily life is organized into patterns and cycles known as *social rhythms*. There are individual differences in how people maintain regular social rhythms for daily activities such as meals, social interactions, sleep, and exercise. According to the social zeitgeber theory (Ehlers 1988), affective disturbances, such as depression, arise from disruptions in social rhythms (i.e., disruptions in the regularity of daily activities), which are often triggered by external events that alter *social zeitgebers*. Hence, maintaining a regular daily routine, especially when it is threatened by external triggers, is critical for emotional well‐being.

Although the social zeitgeber theory was initially proposed to explain the onset of major depressive episodes (Ehlers 1988), it has since been widely tested in relation to other mood states, including (hypo)mania and depression in bipolar disorder (e.g., Alloy et al. 2015; Boland et al. 2012; Shen et al. 2008). Importantly, the link between social rhythm irregularity and depressive symptoms has also been observed beyond clinical populations, with prior studies demonstrating significant associations in non‐clinical samples (e.g., Margraf et al. 2016; Prigerson et al. 1994; Sabet et al. 2021). Together, these findings suggest that social rhythm irregularity may serve as a general risk factor for mood disturbance in both clinical and non‐clinical populations. Focusing specifically on depression, the present study aimed to clarify the mechanism through which social rhythm irregularity is associated with increased depression in college students. In particular, we examined perceived control as a potential psychological mechanism underlying this relationship.

The social zeitgeber theory (Ehlers 1988) posits that disruptions in social rhythms lead to mood disorders mainly through disturbances in biological circadian rhythms. Reflecting this, most prior research on social rhythm has focused on sleep (e.g., sleep quality, the amount of sleep, and self‐efficacy regarding sleep) as the primary mediator between social rhythm irregularity and mood disorders, including depression (e.g., Brown et al. 1996; Kahawage et al. 2022; Meng et al. 2023; Palagini et al. 2022; Sabet et al. 2021; Takaesu et al. 2017). However, despite this extensive focus on sleep‐related disruptions in both theoretical framework and previous empirical evidence, previous research has also suggested the existence of other mechanisms (e.g., Kahawage et al. 2022; Sabet et al. 2021). For example, Sabet et al. (2021) revealed that sleep‐related variables (i.e., self‐efficacy of sleep, sleep satisfaction, and sleep efficiency) partially, but not fully, mediated the relationship between social rhythm disruption and depression. In addition, Kahawage et al. (2022) found that greater disturbances in social rhythms were associated with increased depression, even when controlling for sleep quality. Hence, our understanding of how irregularities in social rhythms may be linked to depression remains limited. Identifying these underlying mechanisms holds significant clinical implications, as they could serve as additional key intervention targets in supporting individuals with depression in maintaining daily routines.

The current study suggests perceived control as a potential mediator. Perceived control refers to an individual's belief in the ability to regulate their internal states and behaviors, influence their environment, and achieve desired outcomes (Wallston et al. 1987). Monk et al. (1997) argued that a regular lifestyle, with consistent timing for daily activities, enhances event predictability and internal regulation, thereby improving mood. Although perceived control and event predictability are distinct, they are closely related: one feels more predictable about daily events when they feel a greater sense of overall control, and vice versa (e.g., Reich and Zautra 1991). Furthermore, we can easily think of daily situations where social rhythm disruptions may lead to a decrease in perceived control. For example, frequent changes in routine may hinder one's ability to schedule appointments, make commitments, or set personal goals. This difficulty in planning can heighten a sense of being out of control, further contributing to depression. Indeed, Huang et al. (2023) have shown that regular social rhythm is linked to higher levels of general self‐efficacy—the perceived ability to handle daily challenges and meet life's demands—a construct closely related to perceived control.

Given the significant relationship between perceived control and decreased depression, as supported by both theories (e.g., learned helplessness theory, which posits that depression is linked to a perceived lack of situational control; Maier and Seligman 1976) and prior research findings (e.g., Ang and Malhotra 2016; Ghorbani et al. 2008; Tighe et al. 2015; Xue et al. 2023), it is plausible that social rhythm irregularity may be associated with a decreased sense of perceived control, which, in turn, could link to increased depression. Thus, this study aimed to examine the mediating role of perceived control in the relationship between social rhythm irregularity and depression. As the social zeitgeber theory identifies sleep‐related disturbance as the primary mechanism (Ehlers 1988), we also sought to examine whether the mediating role of perceived control would remain when sleep quality—one of the most studied mediators in the social rhythm literature—was considered, thereby testing whether perceived control may account for an additional pathway linking social rhythm irregularity and depressive symptoms.

To achieve these aims, we considered several factors in this study. First, we tested these hypotheses using college students, who may be particularly vulnerable to irregular lifestyles due to their greater flexibility in setting schedules, compared to younger individuals (who typically follow fixed school schedules) and older groups, such as workers (who often adhere to routine work schedules). Second, because the social zeitgeber theory (Ehlers 1988) posits that changes in social rhythm can occur after important external events, we conducted this study during the mid‐term and final exam weeks when students often experience changes in their schedules. Third, we tested the model both at the between‐person and within‐person levels, using a 14‐day daily diary study. Although the depression and social rhythm irregularity link at the between‐person level (i.e., those who maintain more regular social rhythm are more likely to report decreased depression) has been studied (e.g., Kahawage et al. 2022; Malkoff‐Schwartz et al. 2000; Margraf et al. 2016; Velten et al. 2018), it remains unclear whether having a more irregular day than usual is also associated with an increase in depressive affect at the end of the day at the within‐person level.

Lastly, we measured social rhythm irregularity using two different methods following the literature: one focusing on the timing of activities (Social Rhythm Metric‐5, SRM‐5; Monk et al. 1991; Monk et al. 2002) and the other on perceived daily irregularity (Brief Social Rhythm Scale, BSRS; Margraf et al. 2016). Irregularity in the SRM is assessed by the exact time an activity occurs each day for a period of time (e.g., An activity is considered regular if, on the days it occurs within a week, it consistently takes place within a 45 min window of its typical time). In contrast, the BSRS assesses individuals' perceived regularity of daily activities (e.g., going to bed, getting out of bed, eating, and meeting others), since some activities may not occur at the same time each day but can still be considered regular. Both measures have been widely used in prior research on depression (e.g., Alloy et al. 2015; Lin et al. 2020 for the SRM variants; Margraf et al. 2016; Sabet et al. 2021 for the BSRS; Swartz et al. 2021 for both measures). Further, there is no consensus on whether irregularity should be measured based on exact timing or subjectively perceived regularity. Therefore, this study used both methods to measure social rhythm irregularity, particularly at the between‐person level.

Hence, based on theories (i.e., the social zeitgeber theory and the learned helplessness theory), we sought to examine concurrent relationships between social rhythm irregularity, perceived control, and depression. Specifically, we examined whether greater irregularity of daily activities during exam periods—a potentially disruptive external event for college students—predicted depressive symptoms during these times via decreased perceived control at the between‐person level, and whether daily social rhythm irregularity predicted end‐of‐day depressive affect via perceived control at the within‐person level. Our specific hypotheses were as follows:

Hypothesis #1 We hypothesized that perceived control would mediate the positive relationship between social rhythm irregularity and depressive symptoms at the between‐person level.

Hypothesis #2 Additionally, perceived control would remain as a significant mediator at the between‐person level, even when sleep quality was included as a simultaneous mediator.

Hypothesis #3 Lastly, we predicted that perceived social rhythm irregularity would be associated with greater depressive affect at the end of the day through decreased daily perceived control over the day at the within‐person level. We only measured perceived social rhythm irregularity due to the lack of a valid method to measure daily irregularity based on exact timing.

For models at the between‐person levels (Hypotheses #1 and #2), we tested both models using the SRM‐5 and BSRS scores, separately. Furthermore, baseline preference for daily routines and trait negative affect, as well as age and gender, were additionally controlled for in all models, as they were associated with depressive symptoms (e.g., Bergua et al. 2006 for preference for daily regularity; Bradley et al. 2011 for trait negative affect).

## 1 Materials and Methods

### 1.1 Participants

Participants were recruited through the psychological research participation pool at Sungkyunkwan University in South Korea. Using G*Power version 3.1.9 (Faul et al. 2007), a priori power analysis was conducted to determine the minimum required sample size for detecting a significant effect in a linear multiple regression model with seven predictors (depression, perceived control, and control variables [sleep quality, preference for routines, negative affect, age, and gender]). We set the most conservative model, including all possible control variables. Assuming a medium effect size (*f* ^2^ = 0.182) based on Sabet et al. (2021) and a *⍺* level of 0.05 with desired power of 0.80, the power analysis indicated that a minimum sample size of *N* = 87 is required to test our hypothesis. Considering an anticipated attrition rate of approximately 20% during the 14‐day study, we aimed to recruit at least 110 participants.

This survey consisted of three time points: the BSRS was measured at baseline (Time 1, T1), followed by a 14‐day daily survey period that included the SRM‐5 (T2), and perceived control and depressive symptoms were measured in the final survey (T3). A total of 124 college students participated in this study (*M* = 19.66 years, SD = 1.31, female = 81.5%). Among those who completed the baseline survey, 101 participants also completed the final survey, and 75 participants completed surveys across all three time points. Because the PROCESS macro used to test indirect effects in the between‐person mediation models handles missing data only through listwise deletion (Hayes 2022), we used data from those with complete data for each model.^1^ Specifically, because all main variables were measured at baseline and the final survey for the models with the BSRS, data from 101 participants were used. For the models using the SRM‐5, which required complete data across all three time points, data from 75 participants were used. In contrast, data from all 124 individuals were included in the within‐person model, in which missing data were handled using maximum‐likelihood estimation. This study was not pre‐registered, and the data used in this study are openly available on the Open Science Framework at https://osf.io/vudt8/?view_only=d612a3f5e851414e83ce1ee894dafeca.

### 1.2 Measures

#### 1.2.1 Social Rhythm Irregularity

To measure social rhythm irregularity at the between‐person level, the SRM‐5 and the BSRS were used. The SRM‐5 is a daily diary‐based questionnaire designed to measure the extent of daily irregularity in an individual's life based on the timing of event occurrences (Monk et al. 2002). It includes five specific events (i.e., “get out of bed,” “have dinner,” “start work, school, housework, volunteer activities, child or family care,” “go to bed,” “first contact with another person”). These activities in the SRM‐5 were selected to capture the regularity of structured routines, which function as social zeitgebers, and they were grouped together based on their functional role rather than their specific content (Monk et al. 1990, 1991). Participants were asked to report the starting time of each of the five events that occurred daily over a 14‐day period. For the item in which multiple activities were grouped together (“start work, school, housework, volunteer activities, child or family care”), participants reported the starting time of the first activity among them. For example, if a person began work at 1:00 p.m. and later engaged in housework on the same day, they would report 1:00 p.m. for this item. Prior work has shown that SRM‐5 scores are strongly correlated with those on the original 17‐item SRM (Monk et al. 2002), providing evidence for its validity.

We followed the scoring algorithm proposed by Monk et al. (1991) (see Monk et al. 1991, for more details). The SRM‐5 score was computed using the following procedures: (1) the average time for a specific activity over each 7‐day period of the 2 weeks was calculated. (2) Outliers, defined as values falling outside 1.5 standard deviations from the mean, were excluded. (3) Using the remaining non‐outlier data, the mean was recalculated, referred to as the habitual time. As a result, a total of five habitual times (one per activity) were computed for each activity. (4) The timing of each activity was recorded as a “hit” if it occurred within 45 min of the individual's habitual time for that activity. Hits for each activity ranged from 0 to 7, and activities with three or more hits per week (considered as regularly occurring daily activities) were selected for further computation. (5) The total SRM‐5 score was calculated by dividing the total number of hits from activities with three or more hits by the number of activities with possible three or more hits. (6) Finally, since the SRM‐5 score is calculated on a weekly basis, the average of the two SRM‐5 scores were used in this study. In the original scale, a higher score indicates regular social rhythms. To align with our hypotheses on social rhythm irregularity, we reverse‐coded the score so that a higher score indicates greater irregularity.

The BSRS is a 10‐item self‐reported questionnaire that evaluates the perceived regularity of daily activities on weekdays and weekends (Margraf et al. 2016). Participants rated their perceived regularity of daily activities (e.g., “going to bed Mondays through Fridays,” “meeting other people at school or work on the weekend”) on a 6‐point Likert scale (1 = very regular, 6 = very irregular), with higher average scores indicating greater irregularity (*α* = 0.72 in this sample).

#### 1.2.2 Perceived Control

The Pearlin Mastery Scale (PMS; Pearlin and Schooler 1978), a 7‐item self‐report questionnaire, was used to assess participants' sense of perceived control (e.g., “I have little control over things that happen to me”). Participants were asked to rate the extent to which they agree with each statement, on a 4‐point Likert scale (1 = strongly disagree, 4 = strongly agree), with higher scores reflecting greater levels of perceived control (*α* = 0.79 in this sample).

#### 1.2.3 Depression Symptom Severities

The Patient Health Questionnaire‐9 (PHQ‐9; Kroenke et al. 2001), a 9‐item self‐report measure, was used to assess the severity of depression symptoms (e.g., “Feeling down, depressed, or hopeless”) over the past 2 weeks. Participants reported how often they had bothered by each depressive symptom in the past 2 weeks on a 4‐point Likert scale (0 = not at all, 3 = nearly every day), with a greater score indicating higher levels of depressive symptoms (*α* = 0.79 in this sample).

#### 1.2.4 Daily Measures

Perceived social rhythm irregularity, perceived control, and depressive affect were also measured at the daily level to test their relationships at the within‐person level. All daily measures used an 8‐point Likert scale (0 = not at all, 7 = very much). Daily perceived social rhythm irregularity was measured using a single item: “How regular has your daily life been today?.” This score was reverse‐coded so that a greater score indicates higher levels of perceived social rhythm irregularity. Daily perceived control was measured using two items from Ryon and Gleason (2014): “I feel that I have control over the things that happen to me today” and “Today I was able to deal with my problems” ($\omega_{\mathrm{between}}$ = 0.99 and $\omega_{\mathrm{within}}$ = 0.83 in this sample). Participants rated the extent to which they agreed with each statement, and average score was used in the analyses. Finally, end‐of‐day depressive affect was measured using a single item, where participants rated how much they experienced feelings of sorrow, depression, and/or loneliness at the end of the day.

#### 1.2.5 Control Variables

##### 1.2.5.1 Sleep Quality

To measure subjective sleep quality, the Pittsburgh Sleep Quality Index (PSQI; Buysse et al. 1989) was used at the last‐day assessment. The PSQI consists of 19 items grouped into seven components: subjective sleep quality, sleep latency, sleep duration, habitual sleep efficiency, sleep disturbances, use of sleeping medication, and daytime dysfunction experienced over the past month. Each component is rated on or converted to a 4‐point Likert scale (0–3). In this study, we used the total PSQI score based on Buysse et al. (1989), ranging from 0 to 21 (0 = good sleep quality, 21 = bad sleep quality; *α* = 0.66 in the current sample).

##### 1.2.5.2 Preference for Routines

The short form of the Preferences for Routines Scale (PRS‐S; Bergua et al. 2021), a 5‐item self‐report measure, was used to assess participants' baseline preference for routines or their tendency to embrace changes in daily life routines (e.g., “I do not like having to change places for eating or watching television”). Each item was rated on a 5‐point Likert scale (1 = almost never true, 5 = almost always true), with higher scores indicating a greater preference for routines. Due to low internal consistency with all five items in the current sample (*α* = 0.56), we removed two most problematic items (items #3 and #5), resulting in acceptable internal consistency (*α* = 0.64). The sum score of the remaining three items were used as baseline preference for routines in this study.

##### 1.2.5.3 Trait Negative Affect

Negative affect was assessed using the negative affect subscale of the Positive and Negative Affect Schedule (PANAS; Watson et al. 1988). Participants rated the extent to which they experienced each of the 10 negative emotions (i.e., irritable, distressed, ashamed, upset, nervous, guilty, scared, hostile, jittery, afraid) on a 5‐point Likert scale (1 = very slightly or not at all, 5 = extremely; *α* = 0.82 in this sample).

### 1.3 Procedures

Each participant participated in the study either 1 week prior to or during their midterm or final exam weeks. After providing informed consent, participants completed the BSRS, the PRS‐S, and the PANAS, and were briefed on the 14‐day daily diary study at T1. The daily diary survey (T2) began the day after the baseline assessment. Over the next 2 weeks, participants received a text message at 10 p.m. every day containing a survey link (i.e., the SRM‐5 and daily measures). A reminder for the survey was sent at 9 a.m. the following day. Daily surveys completed by 12 p.m. (noon) the following day were considered valid and included in the analysis. The final survey (T3) was sent via text message at 10 a.m. on the morning following the completion of the T2 daily survey. In the final survey, participants completed the PMS, the PHQ‐9, and the PSQI. Upon completion of the final survey, participants received research credits for their participation. This study was approved by Sungkyunkwan University's Institutional Review Board (#2024‐04‐014).

A total of 1696 daily diary surveys were submitted, and no responses were recorded outside the valid time window (i.e., between 10:00 p.m. and 12:00 p.m. the following day). Among these, we identified 25 duplicate submissions in which participants completed the survey twice on the same day, resulting in 1671 valid daily diary surveys included in the analyses. On average, participants completed 96.26% of the daily diary surveys (SD = 7.81%, range = 57.14%–100%).

### 1.4 Statistical Analyses

To test Hypothesis #1, that perceived control would mediate the relationship between social rhythm irregularity and depressive symptoms at the between‐person level, two simple mediation analyses were conducted with the SRM‐5 and BSRS as predictors, respectively. The analyses used the PROCESS macro (Hayes 2022) with bootstrapping, employing 5000 resamples from the data set, in R software version 4.3.3 (R Core Team 2021). Further, two parallel mediation models were tested to examine Hypothesis #2 (whether the mediation of perceived control remains significant even after controlling for sleep quality), again with the SRM‐5 and BSRS as predictors, respectively. In these models, both perceived control and sleep quality were simultaneously considered as mediators. All simple and parallel mediation models at the between‐person level included age, gender, preference for routines, and negative affect as covariates.

Lastly, Hypothesis #3, that perceived social rhythm irregularity of the day predicting end‐of‐day depressive affect via perceived control was tested using a multilevel mediation model to account for the nested structure of the data (daily responses nested within individuals), using the lme4 package (Bates et al. 2015). To obtain the *p* value of the fixed effect, the lmerTest package (Kuznetsova et al. 2017) was used, and the confidence intervals for the indirect effect at the within‐person level were computed using Monte Carlo method (Bauer et al. 2006; MacKinnon et al. 2004). We chose to test concurrent rather than temporal relationships (e.g., whether irregularity at time *t*–2 predicts depressive affect at *t* via perceived control at *t*–1) because our primary research question was whether having a more irregular day than usual predicts end‐of‐day depressive affect and whether this association is mediated by daily perceived control.

In a 1‐1‐1 simple mediation model, depressive affect (DA in the formula below) was entered as an outcome variable with social rhythm irregularity (SRI) as a Level‐1 predictor and perceived control (PC) as a Level‐1 mediator. *TIME*, representing the order in which each daily survey was completed (e.g., 1st, 2nd, 3rd), was included as a covariate to account for potential time‐related effects. All Level‐1 predictors were person‐mean centered. The specific formula used is presented below.

A. Perceived control (M) as an outcome

$$\text{Level}\;1:\;{\mathrm{PC}}_{\mathrm{ij}}=\beta_{0j}+\beta_{1j}{\mathrm{SRI}}_{\mathrm{ij}}+\beta_{2j}{\mathrm{TIME}}_{\mathrm{ij}}+\varepsilon_{0j}$$

$$\text{Level}\;2:\;\beta_{0j}=\gamma_{00}+(\mu_{0j})$$

$$\beta_{1j}=\gamma_{10}+\mu_{1j}$$

$$\beta_{2j}=\gamma_{20}+(\mu_{2j})$$

B. Depressive affect (Y) as an outcome

$$\text{Level}\;1:\;{\mathrm{DA}}_{\mathrm{ij}}=\beta_{3j}+\beta_{4j}{\mathrm{PC}}_{\mathrm{ij}}+\beta_{5j}{\mathrm{SRI}}_{\mathrm{ij}}+\beta_{6j}{\mathrm{TIME}}_{\mathrm{ij}}+\varepsilon_{1j}$$

$$\text{Level}\;2:\;\beta_{3j}=\gamma_{30}+(\mu_{3j})$$

$$\beta_{4j}=\gamma_{40}+\mu_{4j}$$

$$\beta_{5j}=\gamma_{50}+\mu_{5j}$$

$$\beta_{6j}=\gamma_{60}+(\mu_{6j})$$

The *a* (path from the predictor to the mediator), *b* (path from the mediator to the outcome), and *c'* (direct path from the predictor to the outcome) paths were modeled at the within‐person level. In the model with perceived control as outcome, $\gamma_{10}$ represented the fixed effect of *a* pathway. In the model with depressive affect as outcome, $\gamma_{40}$ and $\gamma_{50}$ represent the fixed effects of *b* and *c’* pathways, respectively. In a multilevel mediation model, the indirect effect is not simply the product of *a* and *b*, as in a single‐level simple mediation model, because the effects may be correlated. Therefore, the equation for the indirect effect at the within‐person level is given by *ab* + $\sigma_{\mathrm{ab}}$, where $\sigma_{\mathrm{ab}}$ represents the covariance between *a* and *b* random effects (Kenny et al. 2003). In our model, $\mu_{0j}$, $\mu_{2j}$, $\mu_{3j}$, and $\mu_{6j}$were excluded due to their values being close to zero, which resulted in a convergence warning (Bates et al. 2015). In all analyses, gender, grand‐mean‐centered age, baseline preference for routines, and trait negative affect were included as Level‐2 covariates.

## 2 Results

Owing to limited space in the main text, descriptive statistics and correlations of the variables used in this study are presented in Supporting Information S1: Table S1. About 14% of participants (*N* = 14) reported heightened depressive symptoms, with PHQ‐9 scores above the clinical cut‐off (≥ 10; Kroenke et al. 2001). Results from bivariate correlational analyses indicated that social rhythm irregularity, measured by BSRS, but not the SRM‐5, was significantly associated with greater depressive symptoms and lower perceived control at the between‐person level. Further, daily perceived social rhythm irregularity was significantly associated with lower daily perceived control and greater end‐of‐day depressive affect at the within‐person level (see Supporting Information S1: Table S2 for details).

### 2.1 Hypothesis #1: Perceived Control Mediates the Link Between Social Rhythm Irregularity and Depressive Symptoms at the Between‐Person Level

**Figure 1:**
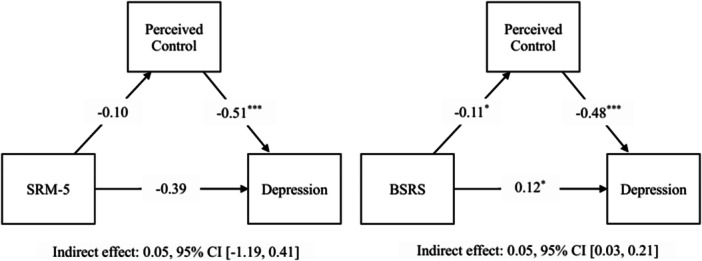
Simple mediation models with perceived control as a mediator. *Note:* Unstandardized path coefficients are reported in the model. *p < 0.05, **p < 0.01, ***p < 0.001. BSRS, brief social rhythm scale; SRM‐5, social rhythm metric‐5.

The results from simple mediation models showed that social rhythm irregularity, measured with the BSRS based on subjective perception, both directly predicted increased depressive symptoms, *b* = 0.12, *β* = 0.22, *p* = 0.011, and indirectly via decreased perceived control, *b* = 0.05, *β* = 0.10, 95% CI [0.03, 0.21]. In contrast, social rhythm irregularity, measured using the SRM‐5 based on exact timing, did not significantly predict depressive symptoms either directly, *b* = −0.39, *β* = −0.10, *p* = 0.332, or indirectly via perceived control, *b* = 0.05, *β* = 0.01, 95% CI [−1.19, 0.41]. The mediation model using the BSRS accounted for 38% of the variance in depressive symptoms, *R*² = 0.38, and the model using the SRM‐5 accounted for 29% of the variance, *R*² = 0.29. Results are presented in Figure 1, and additional details are provided in Supporting Information S1: Table S3.

### 2.2 Hypothesis #2: Perceived Control Remains as a Significant Mediator Even After Controlling for Sleep Quality as Another Mediator

**Figure 2:**
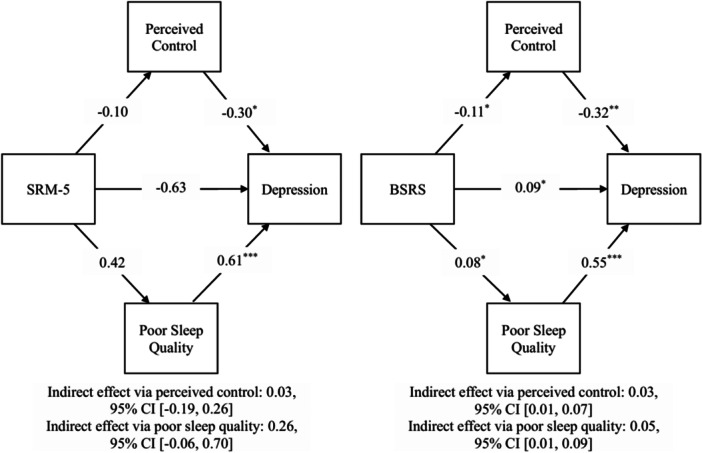
Parallel mediation models with perceived control and sleep quality as mediators. *Note:* Unstandardized path coefficients are reported in the model. *p < 0.05, **p < 001, ***p < 0.001. BSRS, brief social rhythm scale; SRM‐5, social rhythm metric‐5.

Results from two parallel mediation models with perceived control and sleep quality as mediators showed that social rhythm irregularity, measured with the BSRS, significantly predict depressive symptoms directly, *b* = 0.09, *β* = 0.17, *p* = 0.039, indirectly via decreased perceived control, *b* = 0.03, *β* = 0.07, 95% CI [0.01, 0.07], and via increased poor sleep quality, *b* = 0.05, *β* = 0.09, 95% CI [0.01, 0.09]. However, when social rhythm irregularity was measured using the SRM‐5 based on exact timing, it did not significantly predict depressive symptoms either directly, *b* = −0.63, *β* = −0.17, *p* = 0.096, indirectly via perceived control, *b* = 0.03, *β* = 0.01, 95% CI [−0.19, 0.26], or indirectly via sleep quality, *b* = 0.26, *β* = 0.07, 95% CI [−0.06, 0.70]. The parallel mediation model using the BSRS accounted for 50% of the variance in depressive symptoms, *R*² = 0.50, and the model using the SRM‐5 accounted for 42% of the variance, *R*² = 0.42. Results are presented in Figure 2 and details are in Supporting Information S1: Table S4.

### 2.3 Hypothesis #3: Daily Perceived Control Mediates the Link Between Daily Perceived Social Rhythm Irregularity and End‐of‐Day Depressive Affect at the Within‐Person Level

**Figure 3:**
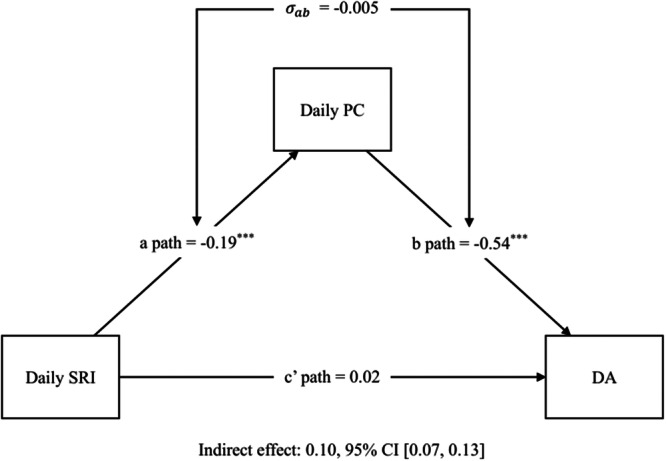
Estimates of the multilevel mediation model with daily perceived control as a mediator. *Note:* Unstandardized path coefficients are reported in the model. *p < 0.05, **p < 0.01, ***p < 0.001. DA, depressive affect at the end of the day; Daily PC, perceived control of the day; Daily SRI, perceived social rhythm irregularity of the day.

Results from the multilevel mediation model revealed that daily perceived social rhythm irregularity significantly predicted daily depressive affect only through increased levels of daily perceived control, $\gamma_{10}\gamma_{40}$ + $\sigma_{\mu_{1j}\mu_{4j}}\;$= 0.10 (indirect effect; *ab* + $\sigma_{\mathrm{ab}}$), 95% CI [0.07, 0.13]. The direct effect was not significant, $\gamma_{50}$ = 0.02 (direct effect; *c’*), *p* < .347 (see Figure 3 and Supporting Information S1: Table S5 for details). As predicted, on days when participants perceived their daily activities as more irregular than average, they also reported decreased perceived control, which, in turn, was linked to more depressive affect at the end of the day. To test whether this mediation is significant even after controlling for baseline social rhythm irregularity, we additionally controlled for the BSRS. The indirect effect remained consistent, $\gamma_{10}\gamma_{40}$ + $\sigma_{\mu_{1j}\mu_{4j}}\;$= 0.10 (indirect effect; *ab* + $\sigma_{\mathrm{ab}}$), 95% CI [0.07, 0.13] (see Supporting Information S1: Table S6 for details).

## 3 Discussion

The present study aimed to examine whether perceived control functions as a significant mediator between social rhythm irregularity and depression, extending prior research that has primarily focused on sleep‐related mechanisms (e.g., sleep quality) proposed in the social zeitgeber theory (Ehlers 1988). Additionally, we extend the investigation to the within‐person level. Results from college students who participated in the study during the exam periods showed that social rhythm irregularity, measured based on one's subjective perception but not exact timing, predicted greater depressive symptoms via lower perceived control. This mediation remained consistent even when sleep quality was added as a parallel mediator. Further, the results from the between‐person level were mirrored at the within‐person level: daily perceived social rhythm irregularity predicted greater depressive affect at the end of the day via decreased perceived control.

The current results support our prediction that perceived control is a significant psychological mediator between disruptions in social rhythms and depression. While previous studies have focused extensively on circadian rhythm‐related variables (e.g., sleep‐related factors) as key mechanisms linking social rhythm disruptions to mood disorders (Ehlers 1988; see Grandin et al. 2006; Haynes et al. 2016), our findings indicate that perceived control—a non‐circadian rhythm psychological factor—also plays a significant mediating role in the relationship between social rhythms and depression. Notably, results from the parallel mediation model showed that perceived control remained significant even when sleep quality was included as another mediator, suggesting that perceived control may represent a distinct pathway linking social rhythm irregularity to affective outcomes and extending beyond the scope of the social zeitgeber theory. Maintaining daily routines despite external disturbances (e.g., exam periods) may function as an effective internal regulation system (Monk et al. 1997) that fosters a sense of predictability and control over daily events. Enhanced perceived control, in turn, can improve mood, as evidenced by the reduced depressive symptoms observed in this study and supported by prior research (see Haynes et al. 2016 for review). This study, along with previous research linking social rhythm to self‐efficacy (Huang et al. 2023), highlights the need to broaden our focus beyond circadian rhythm‐related mechanisms to include other psychosocial mechanisms when investigating the role of social rhythms in depression.

In our sample, social rhythm irregularity measured with the SRM‐5‐based on the exact timing of daily activities—was not significantly associated with depressive symptoms. This contrasts with previous studies that found a significant link between SRM scores and depressive symptoms (e.g., Brown et al. 1996; Shen et al. 2008; Szuba et al. 1992), while aligning with other research that reported a nonsignificant relationship (e.g., Lin et al. 2020). Given that the sample size for our SRM‐5 models (*N* = 75) was smaller than the ideal sample size (*N* = 87) determined by the a priori power analysis, the null findings may reflect insufficient statistical power. However, they may also be attributable to characteristics unique to our sample. Most prior research measuring irregularity with SRM scores has focused on clinical samples (e.g., individuals with bipolar disorder; Shen et al. 2008; Szuba et al. 1992) or those at elevated risk for mood episodes (e.g., bereaved individuals; Brown et al. 1996). It is possible that maintaining routines based on exact timing is more critical for individuals experiencing greater distress, whose rhythms tend to be more disturbed than those of individuals with lower distress (see Grandin et al. 2006). Indeed, Alloy et al. (2015) found that the correlation between SRM scores and depression was significant only among those at heightened risk and not in the lower risk group.

Another possible sample‐related explanation is that our sample consisted exclusively of college students. The SRM conceptualizes social rhythm based on the timing of activities, assuming that activities occurring both frequently and at similar times on the days they occur are regular. However, some individuals have more flexible daily schedules, where activities follow a regular pattern but at varying times. For example, college students often have different class schedules each day, but these schedules repeat weekly, creating a sense of regularity despite daily variations in the exact timing of “going to class.” Supporting this, the correlation between the BSRS and the SRM‐5 was weakly associated in our sample, indicating that they may assess related but distinct constructs. For populations such as college students, social rhythm irregularity may not be best captured by exact timing. This discrepancy underscores the importance of considering measurement approaches that align with individuals' structure of daily schedules. To test this idea, future research with larger samples should further investigate the validity of different social rhythm measures, particularly in populations with diverse lifestyle patterns, to ensure an accurate assessment of social rhythm irregularity.

This study was novel in demonstrating that social rhythm irregularity operates at both the within‐person and between‐person levels. Social rhythm irregularity not only predicts depressive symptoms at the between‐person level but also that social rhythm irregularity of the day predicts depressive affect at the end of the day via decreased daily perceived control at the within‐person level, and such results were significant even after controlling for baseline social rhythm irregularity. These results suggest that even individuals who typically have irregular daily routines may still benefit from maintaining a more regular day. Such routines could foster a sense of control over daily life events, ultimately leading to improved daily affect. It is noteworthy that perceived control fully mediated the relationship between social rhythm irregularity and depressive affect. This finding highlights the important role of perceived control and underscores the potential need to focus on enhancing perceived control when intervening in daily routines to improve depression.

There are several limitations in the current study. First, it employed a partially cross‐sectional design, where depressive symptoms and perceived control (at the between‐person level) were measured simultaneously at T3. Likewise, in the within‐person models, all three variables (daily irregularity, perceived control, and end‐of‐day depressive affect) were measured concurrently. Although our models were grounded in theoretical frameworks (e.g., the social zeitgeber theory), the absence of a clear temporal component in our design limits the ability to draw conclusions regarding the directionality of these associations. Future longitudinal studies—where all three variables are measured at different time points—are necessary to clarify the temporal sequences among these variables, and ideally, experimental research will be required to more directly test the causal pathways proposed by the theory.

The second limitation relates to our measurement. Sleep quality, measured by the PSQI, referred to the past month, which did not align with the 2‐week time frame of the study. Furthermore, because we measured sleep quality only once at T3 rather than on a daily basis, we could not test a parallel mediation model with two simultaneous mediators (sleep quality and perceived control) at the within‐person level. Future research should align the time frames of all key variables and assess daily sleep quality to examine whether perceived control mediates the relationship between daily social rhythm irregularity and end‐of‐day depressive affect, even after accounting for daily sleep quality. In addition, the measures and Likert scales were not identical between the between‐person and within‐person models due to the lack of validated instruments for assessing daily irregularity. Although the overall patterns were consistent, these measurement differences may limit the comparability of findings between the two levels.

The third limitation concerns our sample of non‐clinical college students. On one hand, the relatively flexible schedules of college students, compared to typical workers or youth with more fixed work or school schedules, may have allowed us to better examine the role of social rhythm irregularity in depression, aligning with the theoretical framework indicating that maintaining regularity is particularly important when routines are disrupted (Ehlers 1988). On the other hand, we could not test whether these findings are generalizable to other populations, such as workers with more strictly enforced regular daily schedules, or to clinical samples with depression, who may particularly benefit from this line of research. In addition, we did not assess clinical symptoms beyond depression—such as symptoms of bipolar disorder—so we cannot rule out the possibility that our sample may have exhibited relatively high or low levels of these symptoms, which may have influenced the results.

Lastly, we used “exam periods” as external life events that likely have triggered disruptions in social rhythms. However, we did not test whether all participants experienced actual disruptions in their daily routines during these exam periods. This may also limit the generalizability of the findings to other types of life events. Although prior research has demonstrated a significant relationship between social rhythm irregularity and depressive symptoms even without life events (e.g., Margraf et al. 2016; Sabet et al. 2021), suggesting that maintaining a regular routine is important even on ordinary days, future research should test our hypotheses with more diverse samples and with different types of well‐defined triggering events (e.g., bereavement).

## 4 Conclusions

Our study was novel in testing the mediating role of perceived control, a non‐circadian rhythm‐based factor, in the relationship between social rhythm irregularity and depression at both the between‐person and within‐person levels. The current results extend the social zeitgeber theory by revealing that perceived control plays a significant mediating role even when sleep quality is controlled as a parallel mediator, highlighting the importance of studying other psychological benefits of maintaining regular daily routines. Furthermore, our findings highlight the potential importance of maintaining regular routines to buffer against depression during a stressful life event (exam) among college students. Given that our sample consisted of non‐clinical college students, these findings do not have direct clinical implications for depression. We urge future research to test whether perceived control is a key mechanism linking social rhythm irregularity to depression in clinical populations. If so, interventions targeting social rhythms—such as Interpersonal and Social Rhythm Therapy (IPSRT)—may focus not only on improving sleep but also on enhancing perceived control through more regular daily routines.

## Ethics Statement

This study was approved by Sungkyunkwan University's Institutional Review Board (#2024‐04‐014), and all participants provided informed consent before participating in the study.

## Conflicts of Interest

The authors declare no conflicts of interest.

## Supporting information


**Table S1:** Descriptive Statistics and Bivariate Correlations Among Study Variables at the Between‐Person Level. **Table S2:** Bivariate Correlations Among Daily Measures at the Within‐person Level. **Table S3:** Simple Mediation Models with Perceived Control as Mediator at the Between‐Person Level. **Table S4.:** Parallel Mediation Models with Perceived Control and Sleep Quality as Mediators at the Between‐Person Level. **Table S5:** A Multilevel Mediation Model with Daily Perceived Control as Mediator. **Table S6:** A Multilevel Mediation Model with Daily Perceived Control as Mediator, Controlling for Baseline Social Rhythm Irregularity.

## Data Availability

The data that support the findings of this study are openly available in the Open Science Framework at https://osf.io/vudt8/.

## Endnotes

1 We further considered applying other methods, such as single imputation in conjunction with the PROCESS macro, but did not do so because prior work shows that such methods can bias results (Zhang 2016).

## References

[jclp70124-bib-0001] Alloy, L. B. , E. M. Boland , T. H. Ng , W. G. Whitehouse , and L. Y. Abramson . 2015. “Low Social Rhythm Regularity Predicts First Onset of Bipolar Spectrum Disorders Among At‐Risk Individuals With Reward Hypersensitivity.” Journal of Abnormal Psychology 124, no. 4: 944–952. 10.1037/abn0000107. 26595474 PMC4662076

[jclp70124-bib-0002] Ang, S. , and R. Malhotra . 2016. “Association of Received Social Support With Depressive Symptoms Among Older Males and Females in Singapore: Is Personal Mastery an Inconsistent Mediator?” Social Science & Medicine (1982) 153: 165–173. 10.1016/j.socscimed.2016.02.019. 26907863

[jclp70124-bib-0003] Bates, D. , M. Mächler , B. Bolker , and S. Walker . 2015. “Fitting Linear Mixed‐Effects Models Using lme4.” Journal of Statistical Software 67: 1–48. 10.18637/jss.v067.i01.

[jclp70124-bib-0004] Bauer, D. J. , K. J. Preacher , and K. M. Gil . 2006. “Conceptualizing and Testing Random Indirect Effects and Moderated Mediation in Multilevel Models: New Procedures and Recommendations.” Psychological Methods 11, no. 2: 142–163. 10.1037/1082-989X.11.2.142. 16784335

[jclp70124-bib-0052] Bradley, B. , J. A. DeFife , C. Guarnaccia , et al. 2011. “Emotion Dysregulation and Negative Affect: Association With Psychiatric Symptoms.” Journal of Clinical Psychiatry 72, no. 5: 685–691. 10.4088/jcp.10m06409blu. 21658350 PMC4605672

[jclp70124-bib-0005] Bergua, V. , A. Edjolo , J. Bouisson , C. Meillon , K. Pérès , and H. Amieva . 2021. “Validation of Short Form of Preferences for Routines Scale: Norms in Older Adults.” International Journal of Aging and Human Development 93, no. 2: 767–785. 10.1177/0091415020940213. 32700544

[jclp70124-bib-0006] Bergua, V. , C. Fabrigoule , P. Barberger‐Gateau , J. F. Dartigues , J. Swendsen , and J. Bouisson . 2006. “Preferences for Routines in Older People: Associations With Cognitive and Psychological Vulnerability.” International Journal of Geriatric Psychiatry 21, no. 10: 990–998. 10.1002/gps.1597. 16955425

[jclp70124-bib-0007] Boland, E. M. , R. E. Bender , L. B. Alloy , B. T. Conner , D. R. LaBelle , and L. Y. Abramson . 2012. “Life Events and Social Rhythms in Bipolar Spectrum Disorders: An Examination of Social Rhythm Sensitivity.” Journal of Affective Disorders 139, no. 3: 264–272. 10.1016/j.jad.2012.01.038. 22381951 PMC3368102

[jclp70124-bib-0008] Brown, L. F. , C. F. Reynolds , T. H. Monk , et al. 1996. “Social Rhythm Stability Following Late‐Life Spousal Bereavement: Associations With Depression and Sleep Impairment.” Psychiatry Research 62, no. 2: 161–169. 10.1016/0165-1781(96)02914-9. 8771613

[jclp70124-bib-0009] Buysse, D. J. , C. F. Reynolds , T. H. Monk , S. R. Berman , and D. J. Kupfer . 1989. “The Pittsburgh Sleep Quality Index: A New Instrument for Psychiatric Practice and Research.” Psychiatry Research 28, no. 2: 193–213. 10.1016/0165-1781(89)90047-4. 2748771

[jclp70124-bib-0010] Ehlers, C. L. 1988. “Social Zeitgebers and Biological Rhythms: A Unified Approach to Understanding the Etiology of Depression.” Archives of General Psychiatry 45, no. 10: 948–952. 10.1001/archpsyc.1988.01800340076012. 3048226

[jclp70124-bib-0011] Faul, F. , E. Erdfelder , A. G. Lang , and A. Buchner . 2007. “G* Power 3: A Flexible Statistical Power Analysis Program for the Social, Behavioral, and Biomedical Sciences.” Behavior Research Methods 39, no. 2: 175–191. 10.3758/BF03193146. 17695343

[jclp70124-bib-0013] Ghorbani, N. , S. W. Krauss , P. J. Watson , and D. LeBreton . 2008. “Relationship of Perceived Stress With Depression: Complete Mediation by Perceived Control and Anxiety in Iran and the United States.” International Journal of Psychology 43, no. 6: 958–968. 10.1080/00207590701295264. 22022839

[jclp70124-bib-0014] Grandin, L. D. , L. B. Alloy , and L. Y. Abramson . 2006. “The Social Zeitgeber Theory, Circadian Rhythms, and Mood Disorders: Review and Evaluation.” Clinical Psychology Review 26, no. 6: 679–694. 10.1016/j.cpr.2006.07.001. 16904251

[jclp70124-bib-0015] Hayes, A. F. 2022. Introduction to Mediation, Moderation, and Conditional Process Analysis: A Regression‐Based Approach. 3rd ed. Guilford Press.

[jclp70124-bib-0016] Haynes, P. L. , D. Gengler , and M. Kelly . 2016. “Social Rhythm Therapies for Mood Disorders: An Update.” Current Psychiatry Reports 18: 75. 10.1007/s11920-016-0712-3. 27338753 PMC4919368

[jclp70124-bib-0017] Huang, Q. , X. Wang , Y. Ge , and D. Cai . 2023. “Relationship Between Self‐Efficacy, Social Rhythm, and Mental Health Among College Students: A 3‐Year Longitudinal Study.” Current Psychology 42, no. 11: 9053–9062. 10.1007/s12144-021-02160-1. 34413621 PMC8364412

[jclp70124-bib-0018] Kahawage, P. , B. Bullock , D. Meyer , et al. 2022. “Social Rhythm Disruption Is Associated With Greater Depressive Symptoms in People With Mood Disorders: Findings From a Multinational Online Survey During COVID‐19.” Canadian Journal of Psychiatry 67, no. 11: 832–841. 10.1177/07067437221097905. PMC9096005 35535550

[jclp70124-bib-0019] Kenny, D. A. , J. D. Korchmaros , and N. Bolger . 2003. “Lower Level Mediation in Multilevel Models.” Psychological Methods 8, no. 2: 115–128. 10.1037/1082-989X.8.2.115. 12924810

[jclp70124-bib-0020] Kroenke, K. , R. L. Spitzer , and J. B. W. Williams . 2001. “The PHQ‐9: Validity of a Brief Depression Severity Measure.” Journal of General Internal Medicine 16, no. 9: 606–613. 10.1046/j.1525-1497.2001.016009606.x. 11556941 PMC1495268

[jclp70124-bib-0021] Kuznetsova, A. , P. B. Brockhoff , and R. H. B. Christensen . 2017. “lmerTest Package: Tests in Linear Mixed Effects Models.” Journal of Statistical Software 82, no. 13: 1–26. 10.18637/jss.v082.i13.

[jclp70124-bib-0022] Lin, E. C. L. , M. J. Weintraub , D. J. Miklowitz , et al. 2020. “The Associations Between Illness Perceptions and Social Rhythm Stability on Mood Symptoms Among Patients With Bipolar Disorder.” Journal of Affective Disorders 273: 517–523. 10.1016/j.jad.2020.05.019. 32560948 PMC9012307

[jclp70124-bib-0023] MacKinnon, D. P. , C. M. Lockwood , and J. Williams . 2004. “Confidence Limits for the Indirect Effect: Distribution of the Product and Resampling Methods.” Multivariate Behavioral Research 39, no. 1: 99–128. 10.1207/s15327906mbr3901_4. 20157642 PMC2821115

[jclp70124-bib-0024] Maier, S. F. , and M. E. Seligman . 1976. “Learned Helplessness: Theory and Evidence.” Journal of Experimental Psychology: General 105, no. 1: 3–46. 10.1037/0096-3445.105.1.3.

[jclp70124-bib-0025] Malkoff‐Schwartz, S. , E. Frank , B. P. Anderson , et al. 2000. “Social Rhythm Disruption and Stressful Life Events in the Onset of Bipolar and Unipolar Episodes.” Psychological Medicine 30, no. 5: 1005–1016. 10.1017/S0033291799002706. 12027038

[jclp70124-bib-0026] Margraf, J. , K. Lavallee , X. Zhang , and S. Schneider . 2016. “Social Rhythm and Mental Health: A Cross‐Cultural Comparison.” PLoS One 11, no. 3: e0150312. 10.1371/journal.pone.0150312. 26954568 PMC4783111

[jclp70124-bib-0027] Meng, J. , X. Xiao , W. Wang , Y. Jiang , Y. Jin , and H. Wang . 2023. “Sleep Quality, Social Rhythms, and Depression Among People Living With HIV: A Path Analysis Based on Social Zeitgeber Theory.” Frontiers in Psychiatry 14: 1102946. 10.3389/fpsyt.2023.1102946. 37215662 PMC10192574

[jclp70124-bib-0028] Monk, T. H. , J. F. Flaherty , E. Frank , K. Hoskinson , and D. J. Kupfer . 1990. “The Social Rhythm Metric an Instrument to Quantify the Daily Rhythms of Life.” Journal of Nervous and Mental Disease 178, no. 2: 120–126. 10.1097/00005053-199002000-00007. 2299336

[jclp70124-bib-0029] Monk, T. H. , E. Frank , J. M. Potts , and D. J. Kupfer . 2002. “A Simple Way to Measure Daily Lifestyle Regularity.” Journal of Sleep Research 11, no. 3: 183–190. 10.1046/j.1365-2869.2002.00300.x. 12220313

[jclp70124-bib-0030] Monk, T. H. , D. J. Kupfer , E. Frank , and A. M. Ritenour . 1991. “The Social Rhythm Metric (SRM): Measuring Daily Social Rhythms Over 12 Weeks.” Psychiatry Research 36, no. 2: 195–207. 10.1016/0165-1781(91)90131-8. 2017534

[jclp70124-bib-0031] Monk, T. H. , C. F. Reynolds , D. J. Kupfer , C. C. Hoch , J. Carrier , and P. R. Houck . 1997. “Differences Over the Life Span in Daily Life‐Style Regularity.” Chronobiology International 14, no. 3: 295–306. 10.3109/07420529709001421. 9167890

[jclp70124-bib-0032] Palagini, L. , M. Miniati , D. Marazziti , L. Massa , L. Grassi , and P. A. Geoffroy . 2022. “Circadian Rhythm Alterations May Be Related to Impaired Resilience, Emotional Dysregulation and to the Severity of Mood Features in Bipolar I and II Disorders.” Clinical Neuropsychiatry 19, no. 3: 174–186. 10.36131/cnfioritieditore20220306. 35821870 PMC9263680

[jclp70124-bib-0053] Pearlin, L. I. , and C. Schooler . 1978. “The Structure of Coping.” Journal of Health and Social Behavior 19, no. 1: 2–21. 10.2307/2136319. 649936

[jclp70124-bib-0034] Prigerson, H. G. , C. F. Reynolds , E. Frank , D. J. Kupfer , C. J. George , and P. R. Houck . 1994. “Stressful Life Events, Social Rhytms, and Depressive Symptoms Among the Elderly: An Examination of Hypothesized Causal Linkages.” Psychiatry Research 51, no. 1: 33–49. 10.1016/0165-1781(94)90045-0. 8197270

[jclp70124-bib-0035] R Core Team . 2021. “R: A Language and Environment for Statistical Computing.” R Foundation for Statistical Computing, Vienna, Austria. https://www.R-project.org/.

[jclp70124-bib-0036] Reich, J. W. , and A. J. Zautra . 1991. “Analyzing the Trait of Routinization in Older Adults.” International Journal of Aging and Human Development 32, no. 3: 161–180. 10.2190/4PKR-F87M-UXEQ-R5J2. 1829438

[jclp70124-bib-0037] Ryon, H. S. , and M. E. J. Gleason . 2014. “The Role of Locus of Control in Daily Life.” Personality and Social Psychology Bulletin 40, no. 1: 121–131. 10.1177/0146167213507087. 24107710

[jclp70124-bib-0038] Sabet, S. M. , N. D. Dautovich , and J. M. Dzierzewski . 2021. “The Rhythm Is Gonna Get You: Social Rhythms, Sleep, Depressive, and Anxiety Symptoms.” Journal of Affective Disorders 286: 197–203. 10.1016/j.jad.2021.02.061. 33735764 PMC8058264

[jclp70124-bib-0039] Shen, G. H. , L. B. Alloy , L. Y. Abramson , and L. G. Sylvia . 2008. “Social Rhythm Regularity and the Onset of Affective Episodes in Bipolar Spectrum Individuals.” Bipolar Disorders 10, no. 4: 520–529. 10.1111/j.1399-5618.2008.00583.x. 18452448 PMC4090015

[jclp70124-bib-0040] Swartz, H. A. , B. L. Rollman , D. C. Mohr , S. Sadow , and E. Frank . 2021. “A Randomized Pilot Study of Rhythms and You (RAY): An Internet‐Based Program for Bipolar Disorder Administered With and Without Clinical Helper Support in Primary Care.” Journal of Affective Disorders 295: 183–191. 10.1016/j.jad.2021.08.025. 34469857 PMC8551063

[jclp70124-bib-0041] Szuba, M. P. , A. Yager , B. H. Guze , E. M. Allen , and L. R. Baxter, Jr. 1992. “Disruption of Social Circadian Rhythms in Major Depression: A Preliminary Report.” Psychiatry Research 42, no. 3: 221–230. 10.1016/0165-1781(92)90114-I. 1496054

[jclp70124-bib-0042] Takaesu, Y. , Y. Inoue , K. Ono , et al. 2017. “Circadian Rhythm Sleep‐Wake Disorders Predict Shorter Time to Relapse of Mood Episodes in Euthymic Patients With Bipolar Disorder: A Prospective 48‐Week Study.” Journal of Clinical Psychiatry 79, no. 1: 2651. 10.1016/j.sleep.2017.11.951. 29286593

[jclp70124-bib-0043] Tighe, C. A. , N. D. Dautovich , and R. S. Allen . 2015. “Regularity of Daily Activities Buffers the Negative Impact of Low Perceived Control on Affect.” Motivation and emotion 39: 448–457. 10.1007/s11031-014-9456-8.

[jclp70124-bib-0044] Velten, J. , A. Bieda , S. Scholten , A. Wannemüller , and J. Margraf . 2018. “Lifestyle Choices and Mental Health: A Longitudinal Survey With German and Chinese Students.” BMC Public Health 18: 632. 10.1186/s12889-018-5526-2. 29769115 PMC5956886

[jclp70124-bib-0045] Wallston, K. A. , B. S. Wallston , S. Smith , and C. J. Dobbins . 1987. “Perceived Control and Health.” Current Psychology 6: 5–25. 10.1007/BF02686633.

[jclp70124-bib-0046] Watson, D. , L. A. Clark , and A. Tellegen . 1988. “Development and Validation of Brief Measures of Positive and Negative Affect: The PANAS Scales.” Journal of Personality and Social Psychology 54, no. 6: 1063–1070. 10.1037/0022-3514.54.6.1063. 3397865

[jclp70124-bib-0047] Xue, S. , Q. Gu , K. Zhu , and J. Jiang . 2023. “Self‐Compassion Buffers the Impact of Learned Helplessness on Adverse Mental Health During COVID‐19 Lockdown.” Journal of Affective Disorders 327: 285–291. 10.1016/j.jad.2023.01.099. 36758873 PMC9907794

[jclp70124-bib-0048] Zhang, Z. 2016. “Missing Data Imputation: Focusing on Single Imputation.” Annals of Translational Medicine 4, no. 1: 9. 10.3978/j.issn.2305-5839.2015.12.38. 26855945 PMC4716933

