# Supplementary material for: Irregularity in Daily Activities Predicts Depression via Reduced Perceived Control: A Daily Diary Study
Source: J Clin Psychol. 2026 Mar 2;82(6):938–47. doi: 10.1002/jclp.70124 (PMC13127845; doi:10.1002/jclp.70124)
Supplement: Supplementary file 1 — Table S1: Descriptive Statistics and Bivariate Correlations Among Study Variables at the Between‐Person Level. Table S2: Bivariate Correlations Among Daily Measures at the Within‐person Level. Table S3: Simple Mediation Models with Perceived Control as Mediator at the Between‐Person Level. Table S4.: Parallel Mediation Models with Perceived Control and Sleep Quality as Mediators at the Between‐Person Level. Table S5: A Multilevel Mediation Model with Daily Perceived Control as Mediator. Table S6: A Multilevel Mediation Model with Daily Perceived Control as Mediator, Controlling for Baseline Social Rhythm Irregularity. [file JCLP-82-938-s001.docx]

**Supplementary Materials**

| **Table S1**  *Descriptive Statistics and Bivariate Correlations Among Study Variables at the Between-Person Level.* | | | | | | | | | | | | |
| --- | --- | --- | --- | --- | --- | --- | --- | --- | --- | --- | --- | --- |
|  | 1 | 2 | 3 | 4 | 5 | 6 | 7 | 8 | 9 | 10 | 11 | 12 |
| 1. Age | 1 |  |  |  |  |  |  |  |  |  |  |  |
| 2. Gender | -.27^**^ | 1 |  |  |  |  |  |  |  |  |  |  |
| 3. SRM-5 | -.10 | -.18 | 1 |  |  |  |  |  |  |  |  |  |
| 4. BSRS | -.08 | -.06 | .22^*^ | 1 |  |  |  |  |  |  |  |  |
| 5. PHQ-9 | .06 | .11 | -.13 | .32^**^ | 1 |  |  |  |  |  |  |  |
| 6. PMS | .07 | -.17 | -.02 | -.29^**^ | -.51^***^ | 1 |  |  |  |  |  |  |
| 7. SRI (Daily Diary) | -.06 | -.04 | .21 | .40^***^ | .38^***^ | -.34^***^ | 1 |  |  |  |  |  |
| 8. PC (Daily Diary) | .09 | -.08 | -.03 | -.23^*^ | -.48^***^ | .51^***^ | -.53^***^ | 1 |  |  |  |  |
| 9. DA (Daily Diary) | -.09 | .12 | -.09 | .13 | .54^***^ | -.50^***^ | .12 | -.48^***^ | 1 |  |  |  |
| 10. PSQI | .02 | .08 | .11 | .25^*^ | .62^***^ | -.46^***^ | .22^*^ | -.32^**^ | .36^***^ | 1 |  |  |
| 11. PRS-S | -.11 | -.12 | -.07 | -.02 | .15 | .01 | -.10 | .09 | .21^*^ | .09 | 1 |  |
| 12. PANAS-Neg | -.21^*^ | .13 | .02 | .07 | .36^***^ | -.33^***^ | .16 | -.24^**^ | .38^***^ | .51^***^ | .20^*^ | 1 |
| *Mean* | 19.66 | N/A | 5.11 | 34.49 | 5.45 | 20.82 | 3.45 | 4.63 | 2.67 | 8.14 | 7.14 | 13.10 |
| *Standard deviation* | 1.31 | N/A | 0.98 | 7.62 | 3.88 | 3.14 | 1.14 | 1.08 | 1.23 | 2.99 | 2.56 | 8.18 |
| *Skewness* | 0.85 | N/A | -0.53 | 0.42 | 1.10 | 0.07 | 0.10 | -0.20 | 0.15 | 0.51 | 0.68 | 0.52 |
| *Kurtosis* | 0.38 | N/A | -0.28 | 0.77 | 1.36 | 0.12 | -0.33 | -0.60 | -0.37 | 0.14 | -0.11 | -0.66 |
| *Note*. Gender (Male = 0, Female = 1); SRM-5 = Social Rhythm Metric-5; BSRS = Brief Social Rhythm Scale; PHQ-9 = Patient Health Questionnaire-9; PMS = Pearlin Mastery Scale; SRI = Social Rhythm Irregularity (aggregated); PC = Perceived Control (aggregated); DA = Depressive Affect (aggregated); PSQI = Pittsburgh Sleep Quality Index; PRS-S = Short Scale of Preferences for Routines; PANAS-Neg = Positive and Negative Affect Schedule-Negative Affect Subscale.  ^*^ *p* < .05. ^**^ *p* < .10. ^***^ *p* < .001. | | | | | | | | | | | | |

| **Table S2**  *Bivariate Correlations Among Daily Measures at the Within-person Level.* | | | |
| --- | --- | --- | --- |
|  | 1 | 2 | 3 |
| 1. Daily SRI | 1 |  |  |
| 2. Daily PC | -.25^***^ | 1 |  |
| 3. DA | .13^***^ | -.43^***^ | 1 |
| *Note*. SRI = Social Rhythm Irregularity; PC = Perceived Control; DA = Depressive affect.  ^***^ *p* < .001. | | | |

| **Table S3**  *Simple Mediation Models with Perceived Control as Mediator at the Between-Person Level* | | | | |
| --- | --- | --- | --- | --- |
|  | *b* | *β* | *t* | *p* |
| ***Outcome: Perceived Control*** |  |  |  |  |
| SRM-5 | -0.10 | -0.03 | -0.29 | .774 |
| Age | 0.17 | 0.08 | 0.64 | .552 |
| Gender | -1.18 | -0.15 | -1.32 | .193 |
| Preference for routines | 0.06 | 0.05 | 0.43 | .670 |
| Trait negative affect | -0.12 | -0.32 | -2.62 | .011 |
|  |  |  |  |  |
| ***Outcome: Depression*** |  |  |  |  |
| SRM-5 | -0.39 | -0.10 | -0.98 | .332 |
| Perceived Control | -0.51 | -0.41 | -3.68 | <.001 |
| Age | 0.28 | 0.10 | 0.95 | .344 |
| Gender | 0.22 | 0.02 | 0.21 | .834 |
| Preference for routines | 0.16 | 0.11 | 1.00 | .323 |
| Trait negative affect | 0.09 | 0.19 | 1.62 | .109 |
| ***Outcome: Perceived Control*** |  |  |  |  |
| BSRS | -0.11 | -0.25 | -2.64 | .010 |
| Age | -0.08 | -0.03 | -0.35 | .727 |
| Gender | -1.11 | -0.13 | -1.37 | .175 |
| Preference for routines | 0.05 | 0.05 | 0.46 | .650 |
| Trait negative affect | -0.12 | -0.30 | -3.11 | .002 |
|  |  |  |  |  |
| ***Outcome: Depression*** |  |  |  |  |
| BSRS | 0.12 | 0.22 | 2.60 | .011 |
| Perceived Control | -0.48 | -0.39 | -4.29 | <.001 |
| Age | 0.55 | 0.19 | 2.21 | .029 |
| Gender | 0.92 | 0.09 | 1.03 | .306 |
| Preference for routines | 0.24 | 0.16 | 1.89 | .062 |
| Trait negative affect | 0.10 | 0.19 | 2.09 | .039 |
| *Note. b* = unstandardized coefficient; *β* = standardized coefficient; Gender (Male = 0, Female = 1); SRM-5 = Social Rhythm Metric-5; BSRS = Brief Social Rhythm Scale; Preference for routines was measured using the short form of preferences for routines; Trait negative affect was measured using the negative affect subscale of positive and negative affect schedule; *N* = 75 for the SRM-5 model; *N* = 101 for the BSRS model. | | | | |
| **Table S4**  *Parallel Mediation Models with Perceived Control and Sleep Quality as Mediators at the Between-Person Level* | | | | |
|  | *b* | *β* | *t* | *p* |
| ***Outcome: Perceived Control*** |  |  |  |  |
| SRM-5 | -0.10 | -0.03 | -0.29 | .774 |
| Age | 0.17 | 0.08 | 0.64 | .522 |
| Gender | -1.18 | -0.15 | -1.32 | .193 |
| Preference for routines | 0.05 | 0.05 | 0.43 | .670 |
| Trait negative affect | -0.12 | -0.31 | -2.62 | .011 |
|  |  |  |  |  |
| ***Outcome: Poor Sleep Quality*** |  |  |  |  |
| SRM-5 | 0.42 | 0.15 | 1.38 | .172 |
| Age | 0.31 | 0.15 | 1.40 | .167 |
| Gender | 0.18 | 0.03 | 0.24 | .812 |
| Preference for routines | -0.05 | -0.04 | -0.38 | .709 |
| Trait negative affect | 0.18 | 0.51 | 4.60 | <.001 |
|  |  |  |  |  |
| ***Outcome: Depression*** |  |  |  |  |
| SRM-5 | -0.63 | -0.17 | -1.69 | .096 |
| Perceived Control | -0.30 | -0.24 | -2.19 | .032 |
| Sleep Quality | 0.61 | 0.46 | 3.86 | <.001 |
| Age | 0.06 | 0.02 | 0.21 | .834 |
| Gender | 0.35 | 0.04 | 0.37 | .712 |
| Preference for routines | 0.18 | 0.12 | 1.20 | .236 |
| Trait negative affect | 0.003 | 0.01 | 0.06 | .956 |
| ***Outcome: Perceived Control*** |  |  |  |  |
| BSRS | -0.11 | -0.25 | -2.64 | .010 |
| Age | -0.08 | -0.03 | -0.35 | .727 |
| Gender | -1.11 | -0.13 | -1.37 | .175 |
| Preference for routines | 0.05 | 0.05 | 0.46 | .650 |
| Trait negative affect | -0.12 | -0.30 | -3.11 | .002 |
|  |  |  |  |  |
| ***Outcome: Poor Sleep Quality*** |  |  |  |  |
| BSRS | 0.08 | 0.21 | 2.39 | .019 |
| Age | 0.32 | 0.15 | 1.62 | .109 |
| Gender | 0.45 | 0.06 | 0.63 | .533 |
| Preference for routines | 0.02 | 0.01 | 0.16 | .877 |
| Trait negative affect | 0.19 | 0.50 | 5.52 | <.001 |
|  |  |  |  |  |
| ***Outcome: Depression*** |  |  |  |  |
| BSRS | 0.09 | 0.17 | 2.09 | .039 |
| Perceived Control | -0.32 | -0.26 | -3.03 | .003 |
| Sleep Quality | 0.55 | 0.43 | 4.54 | <.001 |
| Age | 0.38 | 0.13 | 1.67 | .098 |
| Gender | 0.84 | 0.08 | 1.04 | .302 |
| Preference for routines | 0.22 | 0.15 | 1.93 | .057 |
| Trait negative affect | 0.01 | 0.02 | 0.16 | .871 |
| *Note. b* = unstandardized coefficient; *β* = standardized coefficient; Gender (Male = 0, Female = 1); SRM-5 = Social Rhythm Metric-5; BSRS = Brief Social Rhythm Scale; Preference for routines was measured using the short form of preferences for routines; Trait negative affect was measured using the negative affect subscale of positive and negative affect schedule; *N* = 75 for the SRM-5 model; *N* = 101 for the BSRS model. | | | | |

| **Table S5**  *A Multilevel Mediation Model with Daily Perceived Control as Mediator.* | | | |
| --- | --- | --- | --- |
|  | Estimate | *t* | *p* |
| ***Outcome: Perceived Control*** |  |  |  |
| $\gamma_{00}$: fixed intercept | 4.60 | 37.03 | <.001 |
| $\gamma_{10}$: fixed effect of daily SRI | -0.19 | -7.48 | <.001 |
| $\gamma_{20}$: fixed effect of time | 0.01 | 0.94 | .347 |
| Age | 0.04 | 1.18 | .239 |
| Gender | -0.04 | -0.33 | .743 |
| Preference for routines | 0.06 | 3.69 | <.001 |
| Trait negative affect | -0.04 | -6.94 | <.001 |
|  |  |  |  |
| ***Outcome: Depressive Affect*** |  |  |  |
| $\gamma_{30}$: fixed intercept | 2.80 | 22.39 | <.001 |
| $\gamma_{40}$: fixed effect of daily PC | -0.54 | -13.42 | <.001 |
| $\gamma_{50}$: fixed effect of daily SRI | 0.02 | 0.94 | .347 |
| $\gamma_{60}$: fixed effect of time | -0.05 | -4.99 | <.001 |
| Age | 0.03 | 0.75 | .451 |
| Gender | 0.30 | 2.71 | .007 |
| Preference for routines | 0.08 | 4.62 | <.001 |
| Trait negative affect | 0.05 | 9.89 | <.001 |
| *Note.* Gender (Male = 0, Female = 1); SRI = Social Rhythm Irregularity; PC = Perceived Control; Preference for routines was measured using the short form of preferences for routines; Trait negative affect was measured using the negative affect subscale of positive and negative affect schedule. | | | |

| **Table S6**  *A Multilevel Mediation Model with Daily Perceived Control as Mediator, Controlling for Baseline Social Rhythm Irregularity.* | | | |
| --- | --- | --- | --- |
| Parameter descriptions | Estimate | *t* | *p* |
| ***Outcome: Perceived Control*** |  |  |  |
| $\gamma_{00}$: fixed intercept | 4.66 | 37.59 | <.001 |
| $\gamma_{10}$: fixed effect of daily SRI | -0.19 | -7.51 | <.001 |
| $\gamma_{20}$: fixed effect of time | 0.01 | 0.87 | .382 |
| Age | 0.02 | 0.58 | .560 |
| Gender | -0.10 | -0.92 | .360 |
| Preference for routines | 0.05 | 3.24 | .001 |
| Trait negative affect | -0.03 | -6.51 | <.001 |
| BSRS | -0.03 | -5.45 | <.001 |
|  |  |  |  |
| ***Outcome: Depressive Affect*** |  |  |  |
| $\gamma_{30}$: fixed intercept | 2.76 | 22.13 | <.001 |
| $\gamma_{40}$: fixed effect of daily PC | -0.54 | -13.44 | <.001 |
| $\gamma_{50}:$fixed effect of daily SRI | 0.02 | 0.81 | .421 |
| $\gamma_{60}$: fixed effect of time | -0.05 | -4.97 | <.001 |
| Age | 0.04 | 1.16 | .247 |
| Gender | 0.34 | 3.11 | .002 |
| Preference for routines | 0.08 | 4.95 | <.001 |
| Trait negative affect | 0.05 | 9.61 | <.001 |
| BSRS | 0.02 | 3.68 | <.001 |
| *Note.* Gender (Male = 0, Female = 1); SRI = Social Rhythm Irregularity, PC = Perceived Control; BSRS = Brief Social Rhythm Scale; Preference for routines was measured using the short form of preferences for routines; Trait negative affect was measured using the negative affect subscale of positive and negative affect schedule. | | | |
